# Advancing health equity for Indigenous peoples in Canada: development of a patient complexity assessment framework

**DOI:** 10.1186/s12875-024-02362-z

**Published:** 2024-04-29

**Authors:** Anika Sehgal, Rita Henderson, Adam Murry, Lynden (Lindsay) Crowshoe, Cheryl Barnabe

**Affiliations:** 1 Department of Family Medicine, Cumming School of Medicine, University of Calgary, 3330 Hospital Dr NW, Calgary, AB T2N 4N1 Canada; 2 Department of Psychology, University of Calgary, 2500 University Dr NW, Calgary, AB T2N 1N4 Canada; 3 Department of Medicine, Cumming School of Medicine, University of Calgary, 3330 Hospital Dr NW, Calgary, AB T2N 4N1 Canada

**Keywords:** Health equity, Social determinants of health, Health care disparities, Health economics, Primary care

## Abstract

**Background:**

Indigenous patients often present with complex health needs in clinical settings due to factors rooted in a legacy of colonization. Healthcare systems and providers are not equipped to identify the underlying causes nor enact solutions for this complexity. This study aimed to develop an Indigenous-centered patient complexity assessment framework for urban Indigenous patients in Canada.

**Methods:**

A multi-phased approach was used which was initiated with a review of literature surrounding complexity, followed by interviews with Indigenous patients to embed their lived experiences of complexity, and concluded with a modified e-Delphi consensus building process with a panel of 14 healthcare experts within the field of Indigenous health to identify the domains and concepts contributing to health complexity for inclusion in an Indigenous-centered patient complexity assessment framework**.** This study details the final phase of the research.

**Results:**

A total of 27 concepts spanning 9 domains, including those from biological, social, health literacy, psychological, functioning, healthcare access, adverse life experiences, resilience and culture, and healthcare violence domains were included in the final version of the Indigenous-centered patient complexity assessment framework.

**Conclusions:**

The proposed framework outlines critical components that indicate the presence of health complexity among Indigenous patients. The framework serves as a source of reference for healthcare providers to inform their delivery of care with Indigenous patients. This framework will advance scholarship in patient complexity assessment tools through the addition of domains not commonly seen, as well as extending the application of these tools to potentially mitigate racism experienced by underserved populations such as Indigenous peoples.

**Supplementary Information:**

The online version contains supplementary material available at 10.1186/s12875-024-02362-z.

## Introduction

Indigenous peoples in Canada include First Nations, Métis*,* and Inuit peoples, who are the descendants of original inhabitants of the territories claimed in the British North American Act; these peoples now represent 5% of the total country’s population [1, 2], exceeding the growth rate of the non-Indigenous population [3]. Preceding European settlers, Indigenous peoples had established sophisticated self-governing nations reflecting their distinct cultures and diverse languages [4, 5]. Prior to European contact, in general, Indigenous peoples had good health buffered by active ways of life and balanced, nutritional diets that promoted longevity [6–8]. Colonization was an ethnic and cultural genocide with devastating impacts that have persisted until the present-day [9–11]. Calculated practices and policies such as land displacement, forced removal of children from their communities, and the spread of novel and deadly diseases by European settlers extinguished many Indigenous communities and burdened those who survived [12–17], undermining their Ways of Being, Doing, and Knowing [18, 19]. Ample research has linked the longstanding impacts of colonialism directly to the burden of disease and poverty that is experienced by Indigenous peoples today [20–22]. The current study seeks to extend beyond profiling such impacts to identify possibilities for orienting healthcare providers (HCPs) to better respond to the resulting health inequities.

Though Canada has now embarked on a journey of reconciliation with Indigenous peoples to acknowledge the past and its present-day impacts [23, 24], the consequences of forced assimilation cannot be entirely undone and are most evident within the vast health inequities that exist between Indigenous and non-Indigenous peoples [25–27]. In addition to significantly lower life expectancies than their non-Indigenous counterparts [28, 29], Indigenous peoples are disproportionately burdened by diabetes [30, 31], problem substance use [32, 33], perinatal health inequities [34], arthritis [35], and mental health concerns [36, 37] among many other health problems [20, 38]. While persistent disparities attributed to historical consequences have shaped the health of many Indigenous peoples [39, 40], current healthcare systems continue to amplify these inequities through entrenched and systemic racism, played out in discrimination, unequal treatment, and outright violence towards Indigenous peoples [41, 42]. Racism has been described as an “epidemic” within Canadian healthcare systems and a significant contributor to poorer health outcomes by means of stress and harm arising from discriminatory interactions [43–45]. Racial discrimination is also evident in the foundation of healthcare systems, which operate on a Western biomedical epistemology of health that tends to situate disease within the physical bodies of individual patients [46–49], dismissing repercussions of a colonial legacy impacting genetically, culturally, and geographically diverse populations with a shared experience of oppression from colonization [50, 51].

With healthcare systems denying Indigenous peoples’ needs and priorities [52–54], Indigenous patients’ become viewed increasing through the lens of having “complex health needs” [55, 56]. Factors that contribute to this “complexity” are rooted in a legacy of colonization yet often go unnoticed in clinical settings, creating discordance between Indigenous patients and their HCP, as well as discordance between Indigenous patients and broader health systems [57, 58]. Although there is no universal and agreed-upon definition of patient complexity and/or health complexity (terms often used interchangeably within the literature) core indicators include higher healthcare resource utilization, increased need of support, higher risk of adverse health outcomes, and lower satisfaction with care [59, 60]. It is however agreed that patient complexity is not simply co- or multi-morbidity which is marked by the presence of two or more diseases [61]. While co- and/or multi-morbidity may cause a patient to present as “more” complex than patients with a single disease, as in defying easy resolution to their conditions, it is not the only factor that causes a patient to present as complex [61].

Patient complexity is deemed to arise from a patient’s socioeconomic status, environmental and mental health factors, along with the coordination of care and medical decision making which can all complicate a patient’s diagnosis and/or course of treatment causing complexity to arise [62, 63]. Identified domains of patient complexity include:

“demographics (e.g., age, sex, race, and culture), patient personal characteristics or behavior (e.g., communication, burden of disease, coping strategies, and resilience), socio-economic factors, medical, and mental health (e.g., severity of illness, psychiatric disorders, addiction, cognitive impairment), patient risk of mortality, and healthcare system (e.g., care coordination and healthcare utilization), medical decision-making, and environment (e.g., pollution and neighborhood).” [62]

While the conceptualization of patient complexity may be novel to the Western biomedical understandings of health, it serves to reaffirm that broader social factors external to individuals and their physical bodies remain largely unexplored in clinical practice [64].

Given that health inequities across large populations, such as Indigenous peoples, can translate into a higher burden on healthcare systems by way of taxing limited available resources [65], identifying and acting on patient complexity to improve care may feasibly promote better resource allocation to meet patient needs. Patients with complexity often require interventions that are beyond the scope of typical biomedical care and the training of most HCPs [63], which is compounded by healthcare systems being ill-equipped to provide HCPs the appropriate resources necessary to care for patients [64].

Identifying patient complexity is increasingly important, therefore a variety of instruments exist to identify and address patient complexity within different healthcare settings, outpatient or in-patient (including hospitals, treatment centers, and long-term care facilities). Patient complexity assessment tools (PCATs) have emerged as means to aid HCPs in collecting vital information to more effectively deliver care [66–69]. PCATs may provide a comprehensive assessment that considers all aspects of a patient’s needs, and they are inclusive of the patient's experience of their own health [66–68, 70]. PCATs have been noted to enhance patient engagement, more accurately identify the source of complexity challenging care plans, and allow HCPs to engage in appropriate courses of action (e.g. referrals), to improve the health of a patient with complexity [66–68, 70].

PCATs follow two common formats, these being: i) a face-to-face interview between a patient and provider where the provider determines the responses within the PCAT, or ii) for patients to complete in written or digital form on their own in a self-assessment method [65]. PCATs may be specific to in-patient or outpatient populations, and in the case of some populations such as the elderly, PCATs may be applicable to those outside of healthcare settings too [65, 71, 72]. Though PCATs present great utility in identifying patient needs, sources of their complexity, and their resources, limited longitudinal data supports their utility beyond these. Few studies have investigated associations between a patient’s complexity and their subsequent healthcarerelated costs [73–75] and impacts on HCPs [76].

Despite utility for general patient populations, existing PCATs remain inadequate to effectively address the needs of Indigenous patients, as factors most relevant to Indigenous populations often remain under-explored [55]. HCPs rarely understand the full scope of the contributors of poor health that arise from colonial traumas and the impacts of structural inequities that continue to influence the health of Indigenous peoples [50, 77]. Recognizing this gap, the current research aims to identify components that are critical to include in a PCAT developed for use with Indigenous patients. A program of research was undertaken to a) determine the extent to which existing PCATs contain domains for inquiry relevant to the care of Indigenous patients [55], b) explore the components of health complexity among Indigenous patients and the circumstances that allow it to persist [56], and c) identify the most effective constructs that characterize these complexities. The present study aims to engage a diverse panel of healthcare experts to reach consensus on which domains, concepts, and items are critical to effectively assess health complexity among Indigenous patients. Concepts are defined as constructs that represent a logical category while items are those which measure something. This work describes the development of an Indigenous-centered patient complexity assessment framework. The framework will then ultimately be used to derive an appropriate PCAT.

## Methods

### Positionality

The first author, AS, is a settler woman who completed this study as part of her PhD research. The second author, RH, is a settler woman and medical anthropologist who works as an assistant professor and primary care models of care scientist. The third author, AM, is a man of Ukrainian, Irish and Apache descent, and works as an assistant professor of Indigenous psychology. The fourth author, LC, is a Piikani First Nation’s man who works as an associate professor, family physician-scholar and assistant dean, and was a co-supervisor for the first author. The last author, CB, is a Métis woman and a mid-career clinician-scientist in rheumatology and health services research who was the primary supervisor for the first author. As a collective, the team was composed of individuals from both Indigenous and non-Indigenous backgrounds. The authors purposefully approached the research to bring forth unique perspectives with a shared commitment to doing research in a ‘good way’ respecting Indigenous research methodologies and remaining conscious of biases and assumptions.

### Development of core framework structure and candidate item pool

**Fig. 1:**
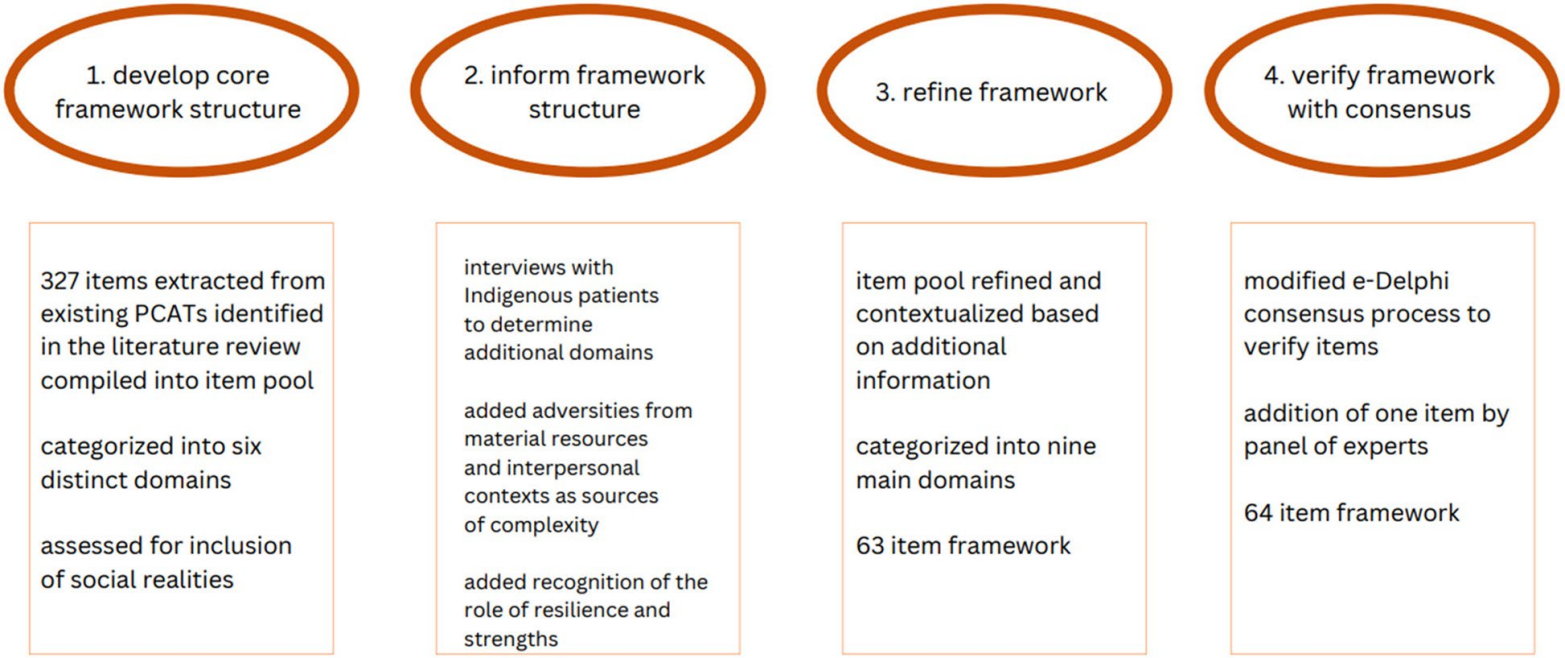
Phases of developing Indigenous-centered patient complexity assessment framework

The framework was developed over the course of four main phases. In the first phase, the core framework structure was identified primarily through review of existing PCATs. In the second phase, the framework was informed by the lived experience of Indigenous patients to embed additional domains relevant to this patient population. In phase three, the framework was refined and categorized logically. Finally, in phase four, the framework was verified with expert consensus. An overview of the phases of this study is presented in Fig. 1.

A scoping review was conducted to identify all existing tools and items, and to assess these in terms of the extent to which they are inclusive of social realities that shape Indigenous health [55, 78]. The review determined that no existing tools are broadly suitable to the needs of Indigenous patients and that existing concepts within many tools need heavy contextualization in order to effectively assess complexity among Indigenous patients [55]. To identify additional domains and concepts of complexity, nine urban Indigenous patients (seven females, two males) were recruited to participate in semi-structured relational conversations with AS to explore the factors contributing to health complexity (see Appendix A for interview guide). Participants were from diverse Indigenous backgrounds but resided in [namewithheld] region where this study took place. The interview guide was pre-tested with the research team (RH, AM, LC, and CB) prior to data collection. Interview data was co-coded with an Indigenous health expert to ensure findings would be culturally sensitive and respect the perspectives of Indigenous peoples. Data was further refined with the research team. Adversity arising from material resources and healthcare interactions were identified as sources of health complexity that elicit psychological responses among patients [56]. Drivers of resilience and other protective factors were also identified that work to prevent health complexity [56]. A targeted search based on additional factors identified through the patient interviews was conducted to identify pre-existing instruments that assess these additional domains to further populate the candidate item pool.

### Refinement and contextualization of framework and candidate item pool

Based on synthesized knowledge from the scoping review and the patient interview study, the third phase of the study outlined here aims to (a) contextualize the most significant emergent concepts to better align with the realities of Indigenous patients; (b) remove redundant items from the pool of candidate items (i.e., concepts and queries for potential inclusion in an Indigenous-focused PCAT); and (c) modify existing items within the concepts in the candidate item pool to better reflect the needs of Indigenous patients. This was done by AS, LC, and CB. Concepts were contextualized (see Table 1) by leveraging a constructivist paradigm [79–81] and building upon the experiences of both LC and CB in their clinical practice with Indigenous patients [82]. This was an iterative, reflective, and time-intensive process which consisted of five meetings over the course of 4 months to review an item pool of over 300 items.

**Table 1:** Indigenous-centered patient complexity assessment framework

Domains and concepts	Conceptualization within an Indigenous context
*Biological domain*	
GENDER IDENTITY AND EXPRESSION	Inquiries into complexity should include both physical sex at birth (important for biological sex considerations of care) but more importantly, one’s gender identity and expression as a potential source of complexity as it is not typically collected in clinic approaches.
SYMPTOMS AND SELF-RATED HEALTH	To address patient complexity, the patient’s perception of the extent to which they are being impaired by their symptoms should be taken into consideration.
PRESENCE OF DISEASES	Patients who present with complexity may have “conditions” rather than diagnosed diseases. Furthermore, patients should have the opportunity to list the conditions that are impacting them; complexity may be due to conditions that are not diagnosed per se yet still have significant impacts on the patient’s capacity to achieve good health.
PRESCRIPTIONS	Inquiries into complexity should include what prescriptions the patient is taking but also the patient’s ability to take those prescriptions as prescribed including whether or not they have coverage to obtain prescriptions.
*Social domain*	
TRANSPORTATION	Complexity assessments should inquire about the mode of transportation used by the patient and the patient’s capacity to reliably access transportation.
CAREGIVER	Inquiries into complexity should include the extent to which a caregiver is required, the availability of a caregiver, and whether or not the patient is a caregiver to someone.
NETWORK	Assessments into complexity should include the patients’ participation in their social network. This is an important indicator of complexity as withdrawal from social networks, along with barriers to being able to participate in a network, can contribute to patient complexity.
HOUSING	Assessments into complexity should include whether or not a patient has a permanent address and if the patient feels they are in a stable housing situation.
DOMESTIC	Assessing one’s domestic environment, including their physical and emotional safety within that environment, is an important social aspect that should be considered when assessing complexity.
INCOME	Assessments into complexity should not only ask about a patient’s financial resources, but also ask where their resources are going or if they have troubles making ends meet despite the income they receive.
FOOD SECURITY	Assessments of complexity should include inquiries into food security and the extent to which a patient has to worry about being able to provide food for themselves consistently.
*Psychological domain*	
MENTAL HEALTH	Inquiries into complexity should include the presence of any mental health concerns but also the extent to which external, social stressors contribute to one’s mental health.
EMOTIONS	A distinction should be made between mental health and the emotions experienced by a patient, including the extent to which one’s emotions have impacted their overall health.
SUBSTANCE USE	Inquiries into the use of substances should be taken into consideration when exploring complexity along with the extent to which those substances are impacting the patient’s health.
*Functioning domain*	
TYPES OF FUNCTIONING	Assessments of functioning should be divided into the types of functioning, including being able to physically function, being able to complete daily tasks, and being able to complete more complex tasks at a higher level of functioning.
*Healthcare access domain*	
OUTPATIENT CARE	The use of outpatient care was examined as an indicator of complexity. Inquiries into complexity should include whether or not a patient is able to access primary health care, the number of visits they average in a year, and the types of specialists they are seeing (if any) for their health.
HOSPITAL USE	Inquiries into complexity should not only include the frequency of hospital visits made by a patient but also how many times a patient was admitted and how many times a patient sought care in the emergency department.
COORDINATION OF CARE	Having adequate coordination of care was identified as an important aspect of managing and preventing complexity. Assessments of complexity should include the extent to which the patient feels the current care they are receiving is sufficient to manage their health.
*Adverse experiences domain*	
ADVERSITIES	Having adverse life experiences was a reality for many Indigenous patients as determined by our data collection. Assessments of patient complexity should include the extent to which one has experienced negative life events that may be contributing to their health to date.
*Healthcare violence domain*	
HEALTHCARE VIOLENCE	Assessments of healthcare violence were missing from inquiry tools yet it emerged as a major cause of complexity as determined by our data collection. Assessments of Indigenous patient complexity should include the extent to which the patient feels their providers and/or systems are capable of addressing their needs in a safe and ethical space.
*Resilience and culture domain*	
RESILIENCE AND CULTURE	From our data collection, resilience, strengths, and connection to one’s culture were identified as “protective factors” that help to mitigate health complexity. Assessments should include inquiries into one’s level of resilience and connection with culture to highlight a strengths-based approach to addressing complexity.
*Section from the HCPs perspective: Biological domain*	
PROGNOSIS	When addressing patient complexity, it is important to ask if all steps have been taken to identify the cause, or if despite taking all steps, there is still uncertainty regarding the cause of the patient’s health concerns.
PRESENCE OF DISEASES	While previously included for patients to list their own diseases and conditions, it is still important for the provider to indicate from their perspective which conditions and diseases are impacting the health of the patient. It is possible that the patient may not always be fully aware of their conditions or they may not accept them and subsequently not list them when asked. Therefore, we included this concept in the provider’s section as well.
*Section from the HCP’s perspective: Social domain*	
DOMESTIC	Assessing one’s domestic environment, including physical and emotional safety within that environment, is an important social aspect that should be considered when investigating complexity. While the patient may report their home situation to be satisfactory, the HCP may deem it to be unfit from their perspective.
*Section from the HCP’s perspective: Literacy*	
GENERAL LITERACY	The provider should take into consideration the patient’s level of general literacy which is required for day-to-day interactions and tasks, including basic reading and writing skills.
HEALTH LITERACY	Health literacy was identified as being unique from general literacy and included the extent to which the patient is able to understand their health conditions and what they need to do in order to manage their health.
*Section from the HCP’s perspective: Healthcare access*	
COORDINATION OF CARE	Having adequate coordination of care was identified as an important aspect of managing and preventing complexity. From the perspective of the HCP, coordination includes the extent to which they feel the current care being received by the patient is adequate (or not).

Redundancy was common within the item pool as several items asked the same questions but with phrasing variations; therefore, these were merged into one item when duplicated. For example, many items asked “what is your age?” or “please indicate your age,” – these were merged into a single item that upheld its core concept to reduce repetition. Existing items were modified as per established recommendations for adaptations of research instruments [83]; they were also adjusted to keep operational equivalence [84], which refers to items kept within similar formats to their original state, including the measurement scale that was used originally [85]. Broader concepts were adjusted to better suit use with an Indigenous population [78, 86]. For example, we broadened the concept of income which commonly asked the patient how much money they made annually to include considerations of the extent to which their money may be going to support others in their household.

### Modified e-Delphi consensus process

The RAND/UCLA appropriateness method [87] was chosen to define the domains and concepts and then refine the item pool. Through a modified e-Delphi consensus process [88–90], concepts were verified to be included in an Indigenous-centered complexity framework along with essential items for collecting information about these concepts. To generate input from diverse perspectives, a purposive panel of HCPs, researchers, and policymakers who work within the field of Indigenous health in Alberta, Canada were identified and invited to participate in this phase. Experts were recruited via email to participate in a 3-round modified e-Delphi consensus process [90] carried out over the course of 4 weeks (October 3rd – November 1st, 2022) using the Qualtrics platform for electronic surveys in the following order:

1. Orientation to the modified e-Delphi consensus process
2. Round #1: voting on concepts
3. Provide a report summarizing results and free-text responses
4. Round #2: voting on items
5. Group discussion
6. Provide a report summarizing results and free-text responses
7. Round #3: voting on items again

Honoraria for experts was not provided therefore only those who felt a commitment towards this work participated. The three rounds progressed attention from identifying concepts, to ranking effectiveness, to discussing and reviewing prioritized items. To launch the e-Delphi consensus process, an introductory orientation meeting was held with the panel of experts to provide an opportunity to ask and address any uncertainties about the process. In Round #1, experts rated *each of the overarching concepts* based on whether or not they *should* be included in a tool of this nature, ranking these on a scale of 1 (absolutely not) to 9 (absolutely yes). Free-text fields were provided for each concept for experts to submit any additional feedback on the concepts and their definitions. Concepts would have to achieve a median score ≥ 7 from the panel with no disagreement among experts to be carried forward into the next rounds. Median scores were calculated according to the RAND/UCLA appropriateness method handbook [87] and disagreement was defined according to the inter-percentile range adjusted for symmetry [87].

In Round #2, experts were asked to rate each item on a scale of 1 to 9 (as above) based on *how effective* [91] the item would be in assessing health complexity with Indigenous patients. Prior to completing responses, expert panel participants were provided via a PDF as an email attachment with the group ratings of concepts from Round #1 as well as the anonymous text feedback from others [92, 93]. An added benefit of sharing the collective responses from prior rounds was that experts could consider the subject matter in terms of how their peers made sense of it. As before, free-text fields were provided for experts to submit any additional feedback on the items and their scoring in Round #2. The inclusion of items in the next round also required median scores ≥7 with no disagreement among experts [87].

Following the completion of Round #2, the experts were invited to a two-hour online video-based group discussion to review concepts and items that were scored lower and to revisit any comments indicating uncertainty or dissension recurring within the free-text fields. This meeting was recorded to track important discussion points and make any final changes or clarifications to the wording and/or scoring of the concepts and items. Changes were made in this group discussion session if the experts indicated consensus amongst themselves, defined as no voiced opposition. The sample of experts consisted of a panel who broadly knew and worked with one another at advanced levels, presuming consensus from no voiced opposition was reasonable. Participants were able to express opposition anonymously by reaching out to the lead author directly.

Following this group session, a report was circulated to all healthcare experts highlighting the summary of suggestions and changes made. A third round of voting was conducted, focused on how effective [91] the experts considered items would be if included in such a tool, again on a scale of 1 (not at all) to 9 (completely). Experts were provided with free-text fields to enter any final feedback on the items. To be included in the final item pool, items once again required median scores ≥7 with no disagreement [87].

### Ethics

This study was approved by the University of Calgary's Conjoint Health Research Ethics Board, Certification #REB20–0972. All participants in the modified e-Delphi consensus process provided their consent to participate in this study.

## Results

### Refinement and contextualization of framework and candidate item pool

After refinement of the framework and candidate item pool, a total of 3 concepts within the biological and social domains were eliminated as they were deemed irrelevant in the context of Indigenous patient complexity. Cognition was eliminated as it was subject to the HCP's perception of a patient's cognitive capacity and therefore deemed potentially dangerous within the context of complexity among Indigenous patients. Weight was eliminated as it only provides one aspect of an individual’s body composition. Community was eliminated as it was not inclusive of what ‘community’ includes for Indigenous peoples, which was rather captured in a domain that was added. Domains of adverse life experiences, healthcare violence, and resilience and culture were added (see Table 2). Items within concepts were also separated if they would be completed by the patient or the HCP, see Appendix B.

**Table 2:** Breakdown of items within each domain and concept included for next phase of research project

Domain and concepts	Number of items
***Biological domain***	***10 in total***
Gender	1 item
Prognosis	3 items
Symptoms and self-rated health	1 item
Presence of disease	2 items
Prescriptions	3 items
Cognition	0 (eliminated concept)
Weight	0 (eliminated concept)
***Social domain***	***15 in total***
Transportation	2 items
Community	0 (eliminated concept)
Caregiver	1 item
Network	3 items
Housing	2 items
Domestic	3 items
Income	3 items
Food security	1 items
***Health literacy domain***	***2 items in total***
General literacy	1 item
Health literacy	1 item
***Psychological domain***	***5 items in total***
Mental health	2 items
Emotions	2 items
Substance use	1 item
***Functioning domain***	***3 items in total***
Physical functioning	1 item
Daily functioning	1 item
Higher-level functioning	1 item
***Healthcare access domain***	***7 items in total***
Outpatient care	3 items
Hospital/ER visits and stays	2 items
Coordination of care	2 items
***Adverse life experiences domain***	***2 items in total***
***Resilience and culture domain***	***11 items in total***
***Healthcare violence domain***	***8 items in total***

### Modified e-Delphi consensus process

The Delphi panel was comprised of *n* = 14 experts in Round #1, *n* = 11 experts in Round #2, and *n* = 10 experts in Round #3. The research team (AS, CB, LC) did not participate in the Delphi panel. There were seven researchers who work in various domains of Indigenous health, three researchers and policymakers in Indigenous health, and four health service providers, including one community health provider, one family physician, one radiation therapist, and one palliative care physician – all of which had extensive experience working with Indigenous patient populations. Of the panel, five members self-identified as Indigenous. A total of *n* = 3 experts attended the summative group discussion session including a community health researcher, an Indigenous health social scientist, and a family physician. In Round #1, all concepts were agreed upon to be included in the Indigenous-centered patient complexity framework (see Table 3).

**Table 3:** Modified e-Delphi consensus process Round #1 voting results

Concepts assessed for inclusion: Patient section	Median rating (IQR)	Concepts assessed for inclusion: HCP section	Median rating (IQR)
***Biological domain***		***Biological domain***	
Gender	8 (7, 8)	Prognosis	8 (8, 9)
Symptoms and self-rated health	9 (8, 9)	Presence of diseases	8 (7, 8)
Presence of diseases	7 (5, 8)	***Social domain***	
Prescriptions	9 (8, 9)	Domestic	6 (5, 7)
***Social domain***		***Literacy domain***	
Transportation	9 (8, 9)	General literacy	7 (6, 8)
Caregiver	9 (8, 9)	Health literacy	8 (7, 8)
Network	8.5 (8, 9)	***Healthcare access domain***	
Housing	9 (9, 9)	Coordination of care	8 (7, 8)
Domestic	9 (8, 9)		
Income	9 (8, 9)		
Food security	9 (8, 9)		
***Psychological domain***			
Mental health	9 (8, 9)		
Emotions	8 (8, 9)		
Substance use	9 (8, 9)		
***Functioning domain***			
Types of functioning	8 (8, 8)		
***Healthcare access domain***			
Outpatient care	8 (8, 9)		
Hospital use	8 (7, 9)		
Coordination of care	8 (7, 8)		
***Adverse experiences domain***			
Adversities	9 (9, 9)		
***Healthcare violence domain***			
Healthcare violence	9 (9, 9)		
***Resilience and culture domain***			
Resilience and culture	9 (9, 9)		

*IQR* nterquartile range

In Round #2, no items were eliminated and all voting members agreed that these items should be included in the proposed Indigenous-centered complexity framework (see Table 4). In the group discussion meeting, items were modified based on wording suggestions and repetitive feedback. One new item was added, which asked whether the patient was a caregiver for someone else for a total of 64 items to be voted on in Round #3. In Round #3, all 64 items met the criteria to be included in the final set of items for an Indigenous-centered PCAT (see Table 4 and Appendix B).

**Table 4:** Modified e-Delphi consensus process Round #2 and #3 voting results

Item assessed for effectiveness	Median rating (IQR) Round #2	Median rating (IQR) Round #3
Q1: Is your gender a source of complexity in your health and healthcare?	7 (7, 8)	8 (7, 8)
Q2: To what degree do your medical condition(s) impact your level of functioning? Explain how?	8 (7, 8)	8 (8, 9)
Q3: List all conditions currently impacting you	8 (8, 9)	9 (8, 9)
Q4: What medications have been prescribed to you?	7 (7, 8)	7.5 (7, 9)
Q5: Do you have cost coverage for the medications that you should be taking?	8 (8, 9)	8 (8, 9)
Q6: In your estimate, how many days of the week do you take your medication as prescribed?	8 (8, 9)	8 (7, 9)
Q7: In terms of transportation, how do you get to the places that you need to go?	8 (7, 9)	8 (7, 9)
Q8: Do you have trouble accessing transportation?	8 (8, 9)	7 (6, 9)
Q9: Do you require a caregiver? If so, do you have one available to you?	8 (8, 8)	8 (8, 8)
Q10: Are you a caregiver for someone else?	*	8 (7, 8)
Q11: Do you have a social network that you participate in?	8 (8, 8)	7.5 (7, 9)
Q12: Are you able to connect with the people closest to you when you want?	7.5 (7, 8)	8 (7, 9)
Q13: How effective is your social network in promoting your health and wellness?	7 (7, 8)	7.5 (7, 8)
Q14: Do you have a permanent address?	8 (5, 9)	8 (7, 8)
Q15: What is your housing situation?	8 (8, 9)	8.5 (8, 9)
Q16: Are you physically safe in your home?	9 (8, 9)	8.5 (8, 9)
Q17: Are you emotionally safe in your home?	8 (7, 9)	8 (8, 9)
Q18: What are your financial resources?	7 (7, 8)	8 (6, 8)
Q19: Do you have troubles making ends meet?	8 (8, 9)	8 (8, 9)
Q20: To what extent is the money that you receive used to support others?	8 (7, 8)	7.5 (7, 8)
Q21: Do you often worry about how you will get your food?	9 (8, 9)	9 (8, 9)
Q22: Do you feel like your mental health impacts your day-to-day life?	7 (7, 9)	8 (8, 8)
Q23: To what extent do social stressors impact your mental health?	8 (8, 9)	8 (8, 8)
Q24: How would you rate your emotional wellness?	7 (7, 8)	8 (7, 8)
Q25: To what extent have your emotions impacted your health and well-being?	8 (7, 8)	8 (8, 8)
Q26: Are you using any substances? If yes, to what extent are these substances influencing your health?	7 (6, 8)	7.5 (7, 8)
Q27: To what extent do health issues impair your physical functioning?	8 (8, 8)	8 (8, 8)
Q28: To what extent are you able to take care of all your personal needs (such as bathing and feeding yourself)?	8 (8, 8)	8.5 (8, 9)
Q29: To what extent are you able to complete household and domestic tasks (such as getting groceries and managing your money)?	8 (8, 9)	8 (8, 9)
Q30: Do you have a primary health care provider?	9 (8, 9)	9 (8, 9)
Q31: Approximately how many times have you visited a doctor and/or nurse practitioner in the past year?	7 (6, 8)	8 (8, 8)
Q32: Are you seeing any specialist doctors? If yes, what types of specialists?	8 (7, 8)	8 (8, 8)
Q33: Approximately how many times in the past year have you been admitted to the hospital for your health condition(s)?	7 (7, 8)	8 (8, 8)
Q34: Approximately how many times in the past year have you visited the ER or urgent care for your health condition(s)?	7 (7, 7)	8 (8, 8)
Q35: Do you feel that the coordination of care you are receiving now is effective for your health needs?	8 (8, 8)	7.5 (7, 8)
Q36: Have any of these things been a worry for you or anyone else living in this house during the last year? Serious illness**/**Serious accident**/**Death of family member or close friend**/**Divorce or separation**/**Not able to get a job**/**Lost job**/**Alcohol related problems**/**Drug related problems**/**Seeing fights or people beaten up**/**Abuse or violent crime**/**Trouble with the police**/**Gambling problem**/**Member of family sent to jail**/**Overcrowding at home**/**Discrimination/Racism**/**Vandalism or Malicious damage to property	8 (7, 9)	8.5 (8, 9)
Q37: How has your childhood history shaped your health and well-being today?	8 (6, 8)	8 (8, 8)
Q38: Feeling that my doctor doesn’t give me clear enough directions on how to manage my conditions	8 (6, 8)	8 (8, 9)
Q39: Feeling that I don’t have a doctor who I can see regularly enough about my conditions	9 (8, 9)	8 (8, 8)
Q40: Feeling that I will end up with serious long-term complications, no matter what I do	7 (6, 9)	8 (8, 8)
Q41: Feeling that my doctor doesn’t know enough about my conditions and its care	8 (8, 9)	8 (7, 8)
Q42: Feeling that my doctor doesn’t take my concerns seriously enough	9 (9, 9)	8.5 (8, 9)
Q43: To what extent do you feel like you’ve been helped by previous healthcare providers?	9 (8, 9)	8 (7, 8)
Q44: Do you feel like you can trust the healthcare system to treat you well and address your health needs?	9 (9, 9)	9 (8, 9)
Q45: Do you feel safe when accessing healthcare services?	9 (9, 9)	9 (9, 9)
Q46: I feel a strong attachment towards my [Aboriginal/FNMI] community or Nation	9 (8, 9)	8.5 (8, 9)
Q47: I have a strong sense of belonging to my [Aboriginal/FNMI] community or Nation	9 (8, 9)	9 (8, 9)
Q48: I feel a strong connection to my ancestors	9 (8, 9)	8 (8, 8)
Q49: I can understand some of my [Aboriginal/FNMI] language	8 (8, 9)	8 (8, 9)
Q50: I have participated in a cultural ceremony (examples: Sweatlodge, Moon Ceremony, Sundance, Longhouse, Feast or Giveaway)	9 (8, 9)	8 (8, 8)
Q51: I have a traditional person, Elder or Clan Mother who I talk to	9 (8, 9)	8 (8, 8)
Q52: When I am physically ill, I look to my [Aboriginal/FNMI] culture for help	8 (8, 9)	8 (8, 8)
Q53: When I am overwhelmed with my emotions, I look to my [Aboriginal/FNMI] culture for help	8 (8, 8)	8 (8, 8)
Q54: Do you feel like you have a balanced state of health?	8 (7, 9)	9 (8, 9)
Q55: Are you able to connect with your culture? If not, do you want to?	9 (9, 9)	9 (8, 9)
Q56: What are your goals in terms of your health? How are you willing to achieve those goals?	8 (7, 8)	8 (6, 9)
***Items assessed by HCP***		
Q57: Thinking about your client’s physical health needs, are there any symptoms or problems (risk indicators) you are unsure about that require further investigation?	8 (8, 8)	8 (8, 9)
Q58: Are further investigations required to understand the patient’s health concerns?	8 (7, 9)	8 (7, 9)
Q59: Are there unexplained symptoms and/or signs despite having completed investigations and consultations?	8 (8, 9)	8 (8, 9)
Q60: List all diagnoses currently impacting the patient	7 (5, 8)	8.5 (8, 9)
Q61: What is their home environment (including domestic violence, insecure housing, neighbor harassment)?	8 (7, 9)	7.5 (6, 8)
Q62: Is the patient able to read and write?	8 (6, 8)	7.5 (7, 8)
Q63: How well do you perceive your patient understands their health and well-being (symptoms, signs or risk factors) and what they need to do to manage their health?	8 (5, 8)	7 (7, 8)
Q64: Is the current coordination of care effective for the patient’s health needs?	8 (7, 8)	8 (8, 9)

*IQR* Interquartile range

^a^Effectiveness as defined by the Health Quality Council of Alberta [91]

*no score available in Round #1 as item was added in Round #2

Within comments in the free-text fields provided and during the group discussion, concerns were raised regarding the feasibility of a tool if it were to include all items, given their length and the lack of item reduction that took place during Rounds #2 and #3. Despite an emphasis on reduction during the group discussion and circulated report to experts, all items met the criteria to be included as part of the final tool, demonstrating their importance in assessing complexity among Indigenous patients and stirring possible need for other strategies to help reduce the burden of length.

## Discussion

Healthcare systems in Canada are set up in ways that tend to dismiss the colonial history and its ongoing impacts on Indigenous peoples’ health [94, 95]. Current models of healthcare delivery seldom take into account broader determinants of health that influence Indigenous peoples and their well-being, in turn, further perpetuating health inequities [94, 96]. Clinical frameworks can serve as tools to foster a culturally safe environment [97, 98], and respectful dialogue [99, 100] with Indigenous patients to promote shared decision-making [101, 102], honour self-determination in health [103, 104], and arrive at mutually agreed-upon management plans that advance good health while simultaneously honouring Indigenous values [105, 106]. This study describes the development of a framework tailored for use with Indigenous peoples in clinical settings, with the intention that it may eventually serve as a resource for HCPs to engage critical theoretical domains important to complex patient care. The goal of the framework is to provide a categorization of the dimensions that are encompassed within complexity—providing an understanding of why a patient may be present as “complex” in healthcare settings. By purposefully exploring the aspects that all collectively contribute to complexity observed in patient presentations, this framework aims to help HCPs gain insight into the nature of health complexity among Indigenous patients, ultimately promoting their capacity to navigate and address the challenges that arise with health complexity. As the culmination of a multi-phased approach, findings offer a theoretical structure for key domains of complexity shaping Indigenous patient health. The present study explores the sources of complexity and their presentations among Indigenous patients.

The Truth and Reconciliation Commission (TRC) of Canada has called for HCPs to be educated on the impacts of colonialism on Indigenous health, to promote cultural safety and sensitivity in healthcare interactions [23, 78, 107]. HCPs today may not fully understand or perceive the historical and ongoing drivers of poor health that continue to harm Indigenous patients, presenting with an overall lack of awareness that can impact the effectiveness of healthcare delivery [47, 108]. Service innovations that exist at the interface of Indigenous patients and HCPs, such as a patient complexity assessment framework, can be a resource to bridge gaps in understanding contributors to good health for Indigenous patients. This framework could provide comprehensive high-quality care provisions by opening possibilities for addressing social and structural determinants of health within Western biomedical spaces.

For Indigenous peoples, health is inextricably tied to the determinants of health that have arisen from colonization. As outlined in the TRC’s 94 Calls to Action [23], there are many possibilities within the health sector to support reconciliation with Indigenous peoples [109]. The findings of this study are aligned with the directions of reconciliation set out by the TRC Calls to Action [23]. By providing care that is better suited to the needs of Indigenous peoples, we can advance health equity [110–112] and diminish the impacts of systemic factors that negatively influence the health of Indigenous peoples. Having appropriate resources, such as an Indigenous-centered patient complexity framework, is a means to foment capacity among HCPs to better engage with Indigenous patients, as well as to acknowledge and act on the insidious role of colonization in shaping health outcomes [113, 114]. HCPs require the awareness and competencies to effectively address complexity among Indigenous patients [107, 113, 115, 116]. The patient complexity assessment framework presented here builds evidence for a potential resource aimed at enabling deeper and more meaningful clinical interactions between HCPs and Indigenous patients. It can help to advance cultural safety in clinical settings which refers to having practices rooted in a basic understanding of Indigenous peoples’ beliefs and history while also engaging a process of self-reflection to understand the power differences between the HCP and patient which can impact the process of care and healing [117]. Not only might the framework provide a lens for HCPs to better discern health determinants stemming from colonization, but it may also enhance the capacity of the HCPs to reconstruct their pathways of care to more effectively address the needs of Indigenous patients. This work supports directions to culturally safe care and leads the way in decolonizing approaches to care.

### Strengths and limitations

The framework presented offers HCPs an opportunity to understand the nature and specific origins of the realities that continue to shape the health of Indigenous patients. This knowledge presents an actionable opportunity to shift HCPs’ tendencies to locate blame within the patient for health outcomes to instead locate cause within structural and systemic dynamics arising from inequity as an outcome of colonization. The approach taken to develop this framework is critical and rigorous in how it engages with many disciplines of knowledge, centering Indigenous knowledge throughout the process. The framework holds significant theoretical rigour that provides a strong foundation of knowledge for informing any future PCATs given its multi-phased development and continuous refinement. Furthermore, items developed are highly validated derived from published measures, informed by the lived experiences of Indigenous patients, contextualized to reflect the social realities that shape the health of Indigenous peoples, and reviewed by healthcare experts within the field of Indigenous health.

As noted in the results section, it is limited by the number of items selected to be included in the proposed Indigenous-centered PCAT, risking that such a tool is burdensome to employ in regular clinical practice. Future advancements of this work will employ psychometric methods and experiment with novel delivery approaches in order to reduce the burden of eliciting items and to ensure the tool’s feasibility for use in clinical settings. Considerations of health literacy will also be undertaken in the refinement of the items as many of them may not be easily understood by patients without prior knowledge and/or clear definitions. Within the item pool, there are no items that directly ask about the role of discrimination, racism, stereotyping, and mistrust in perpetuating complexity within the Indigenous patient. While important concepts, the nature of the tool’s employment causes concern to be cautious in alienating the HCPs and creating context that causes discomfort for both the patient and HCP. Likewise, inquiries about systemic inequities may serve to paralyze HCPs, implicitly suggesting that if complexity is caused by structural and institutional factors, HCPs are then incapable of addressing complexity within the Indigenous patient. This is a limitation of the nature of such tools, and future advancements of this research will explore avenues to create safety for such disclosures. A key question remains whether complexity, which is an unobservable construct, exists as a unitary construct or if it represents a collection of correlated facets without a common core [118]. Future analyses will explore the presence of any dimensions within the framework that reflect a global, underlying construct of complexity [ 118]. Another limitation is that both the Indigenous patients who contributed to phase two of this work and the HCPs, policymakers, and researchers who participated in phase four of this work represent the regional area of Alberta, Canada. Future advancements of this work will explore the applicability of such a PCAT outside of this region.

### Future steps

While we have presented a framework of Indigenous patient complexity, future steps of this work are aligned with addressing the limitations of this study and will advance the goal of having a PCAT for use in clinical settings. A subsequent PCAT developed from the framework presented here could be used in practice as an initial screening tool to assess new Indigenous patients for complexity or as a longitudinal tool that may be employed across many points throughout the patients’ healthcare journey providing opportunity for comparative analyses to determine changes in complexity. Pilot data will be collected for a factor analysis in a bid to reduce the number of items included in such an Indigenous-centered PCAT [119–121]. Using the framework presented as a model of data, we will test hypotheses regarding the number of factors, the correlation between those factors, and the relationship of the items to the factors [119–121]. Factors are larger than items and concepts to allow for refining and rendering a more precise tool. Pilot data will also help to inform the best use of the tool, ensuring Indigenous patient needs are being met.

## Conclusion

Through the critical application of the integrated concepts presented within the framework, we put forth a set of recommendations to improve clinical care interactions between HCPs and Indigenous patients, advance cultural safety in healthcare settings, and hold space for Indigenous epistemologies and experiences within social and healthcare structures that continue to systemically disadvantage Indigenous peoples. The framework presented here offers an evolving body of knowledge to enhance capacity to inform HCPs, systems, and policies on how to facilitate better health outcomes with Indigenous peoples. Future research will work to reduce the number of items in such a tool to advance usability within clinical settings.

### Supplementary Information

**Supplementary Material 1.**

**Supplementary Material 2.**

**Supplementary Material 3.**

## Data Availability

The datasets generated and/or analysed during the current study are not publicly available due privacy considerations but are available from the corresponding author on reasonable request.

## Abbreviations

HCPs: Healthcare providers
PCATs: Patient complexity assessment tools
IQR: Interquartile range
TRC: Truth and Reconciliation Commission
