# Supplementary material for: Advancing health equity for Indigenous peoples in Canada: development of a patient complexity assessment framework
Source: BMC Prim Care. 2024 Apr 29;25:144. doi: 10.1186/s12875-024-02362-z (PMC11057171; doi:10.1186/s12875-024-02362-z)
Supplement: Supplementary file 1 — Supplementary Material 1. [file 12875_2024_2362_MOESM1_ESM.docx]

**Appendix A**

**Interview #1:** Purpose to develop rapport with the patient and serve as an introductory meeting to provide context for next interviews.

**Questions:**

- Tell me about yourself?
- What are 3 words a close friend would use to describe you?
- What is something you are looking forward to when the restrictions around COVID-19 are eased?

**Interview #2:** Purpose to explore what being healthy means, what constitutes health, and what factors help and hinder the patient to be healthy.

**Questions:**

- As an Indigenous person, what does “health” or “being healthy” mean for you and what does it include?
- What are some other things that play a role in you being a healthy individual?
  - How is culture a part of being healthy for you?
  - What about income?
- What are some things that undermine your health or contribute to you not having good health?
  - Does money impact your health?
  - What about your living conditions?
- Is there anything else you would like to share with me today?

**Interview #3:** Purpose to explore healthcare experiences and how their complexity has been addressed (or not) in healthcare settings.

**Questions:**

- Thinking about the physicians who have provided care to you, have you experienced care where one has effectively and respectfully explored your specific complexities?

Has a physician personalized care to you that responded to and fully respected your complex contexts?

- - If not, what would have been a good question for the physician to ask you if they wanted to understand your complexities?
- Thinking now about other staff/nurses/social workers who have provided care to you, have you experienced care where one has effectively and respectfully explored your specific complexities?

For example, has a nurse, social worker, or anyone other than the physician personalized care to you that responded to and fully respected your complex contexts?

- - Are these individuals doing anything differently than the physician to understand the source
- Is there anything else you would like to share with me today?

**Demographic Questions:**

- What is your age?
- What is your gender?
- Do you identify yourself as First Nations, Métis, or Inuit?
