# Supplementary material for: Advancing health equity for Indigenous peoples in Canada: development of a patient complexity assessment framework
Source: BMC Prim Care. 2024 Apr 29;25:144. doi: 10.1186/s12875-024-02362-z (PMC11057171; doi:10.1186/s12875-024-02362-z)
Supplement: Supplementary file 2 — Supplementary Material 2. [file 12875_2024_2362_MOESM2_ESM.docx]

**Appendix B.** Phase three framework concepts and items.

The following questions ask you about various factors that may be impacting your health and contributing to health complexity. Please answer each of the questions to the best of your ability so that your provider can get a “full picture” of what factors may be impacting your health.

| Biological Domain |  |
| --- | --- |
| GENDER: While age and sex demographic questions were a part of identified tools, it was determined to be redundant to collect these demographics in the clinical setting where they are already available. Data collection should include both physical sex at birth (important for biological sex considerations of care) but more importantly, one's gender identity and expression as a potential source of complexity as it is not typically collected in clinic approaches. | |
| Is your gender a source of your health complexity? | Enter text: ________________ |
| SYMPTOMS AND SELF-RATED HEALTH: Several items from identified tools asked about specific symptoms affecting various systems within the patient. To address patient complexity, the patient’s perception of the extent to which they are being impaired by their symptoms is important. | |
| To what degree do your medical condition(s) impact your level of functioning?  Explain how? | \| Extremely \| Moderately \| Mildly \| Not at all \| \| --- \| --- \| --- \| --- \|   Enter text: ________________ |
| PRESENCE OF DISEASES: Items from identified tools asked about the presence of very specific diseases and asked the healthcare provider to indicate these. Patients who present with complexity may have “conditions” rather than diagnosed diseases. Furthermore, patients should have the opportunity to list the conditions that are impacting them; complexity may be due to conditions that are not diagnosed per se yet still have significant impacts on the patient’s capacity to achieve good health. | |
| List all conditions currently impacting you | Enter text: ________________ |
| PRESCRIPTIONS: Items from identified tools included questions that asked what prescriptions the patient was taking but did not assess their capacity to take those prescriptions as prescribed. Data collection regarding prescriptions should include the patient’s ability to take those prescriptions as prescribed including whether or not they have coverage to obtain prescriptions. | |
| What medications have been prescribed to you? | Enter text: ________________ |
| Do you have cost coverage for the medications that you should be taking? | \| Yes \| No \| \| --- \| --- \| |
| In your estimate, how many days of the week do you take your medication as prescribed? | Enter text: ________________ |
| Social Domain | |
| TRANSPORTATION: Identified tools included items that inquired the mode of transportation used by the patient but did not assess the patient’s capacity to reliably access transportation. Data collection should include both the mode of transport used and the reliability of that transportation. | |
| In terms of transportation, how do you get to the places that you need to go? | Enter text: ________________ |
| Do you have trouble accessing transportation? | \| Yes \| No \| \| --- \| --- \| |
| CAREGIVER: Identified tools included items that asked about the presence of a caregiver, the role of the caregiver, and the frequency of care provided by the caregiver. Assessments of complexity should include the extent to which a caregiver is required and furthermore, the availability of a caregiver as some patients may require a caregiver but still do not have one available to them. | |
| Do you require a caregiver? If so, do you have one available to you? | \| Yes \| No \| \| --- \| --- \|  \| Yes \| No \| \| --- \| --- \| |
| NETWORK: Identified tools included items that assessed the patients’ participation in their social network. This is an important indicator of complexity as withdrawal from social networks, along with barriers to being able to participate in a network, can contribute to patient complexity. | |
| Do you have a social network that you participate in? | \| Yes \| No \| \| --- \| --- \| |
| Do you experience barriers in participating in your social network? | \| Yes \| No \| \| --- \| --- \| |
| How effective is your social network in promoting your health and wellness? | \| Extremely \| Moderately \| Mildly \| Not at all \| \| --- \| --- \| --- \| --- \| |
| HOUSING: Identified tools included items that asked whether or not a patient had a permanent address. While this is an important aspect that should be considered, additional items should ask the patient feels they are in a stable housing situation. | |
| Do you have a permanent address? | \| Yes \| No \| \| --- \| --- \| |
| What is your housing situation? | Enter text: ________________ |
| DOMESTIC: Assessing one’s domestic environment, including their physical and emotional safety within that environment, is an important social aspect that should be considered when assessing complexity. | |
| Are you emotionally safe in your home? | \| Yes \| No \| \| --- \| --- \| |
| Are you physically safe in your home? | \| Yes \| No \| \| --- \| --- \| |
| INCOME: Income is another social indicator that was included in identified tools. Items should not only ask about a patient’s financial resources, but also ask where their resources are going or if they have troubles making ends meet despite the income they receive. | |
| What are your financial resources? | Enter text: ________________ |
| Do you have troubles making ends meet? | \| Yes \| No \| \| --- \| --- \| |
| To what extent is the money that you receive used to support others? | Enter text: ________________ |
| FOOD SECURITY: Assessments of complexity should include inquiries into food security and the extent to  which a patient has to worry about being able to provide meals for themselves consistently. | |
| Do you often worry about how you will get your meals? | \| Always \| Often \| Rarely \| Never \| \| --- \| --- \| --- \| --- \| |
| Psychological Domain | |
| MENTAL HEALTH: Identified tools included questions that asked about the presence of mental health concerns and the extent to which they impact the overall health of the patient. Data collection should include the presence of any mental health concerns but also the extent to which external, social stressors contribute to one’s mental health. | |
| Do you have mental health concerns?  If yes, have your mental health concerns impacted your capacity to address your health? | \| Yes \| No \| \| --- \| --- \|  \| Yes \| No \| \| --- \| --- \| |
| To what extent do social stressors impact your mental health? | \| Extremely \| Moderately \| Mildly \| Not at all \| \| --- \| --- \| --- \| --- \| |
| EMOTIONS: While identified tools included questions about a patient’s psychological state, a distinction was made between mental health and the emotions experienced by a patient, including the extent to which one’s emotions have impacted their overall health. | |
| How would you rate your emotional wellness? | \| Very poor \| Poor \| Good \| Excellent \| \| --- \| --- \| --- \| --- \| |
| To what extent have your emotions impacted your health and wellbeing? | \| Extremely \| Moderately \| Mildly \| Not at all \| \| --- \| --- \| --- \| --- \| |
| SUBSTANCE USE: Assessing the use of substances should be included in data collection along with the extent to which those substances are impacting the patient’s mental health and overall wellbeing. | |
| Are you using any substances?  If yes, to what extent are these substances adversely impacting your health and wellbeing? | \| Yes \| No \| \| --- \| --- \|  \| Extremely \| Moderately \| Mildly \| Not at all \| \| --- \| --- \| --- \| --- \| |
| Functioning Domain | |
| TYPES OF FUNCTIONING: Functioning questions were included across the identified tools. Assessments of functioning should be divided into the types of functioning, including being able to physically function, then being able to complete daily tasks, followed by higher level functioning to complete more complex tasks. | |
| To what extent do health issues impair your physical functioning? | \| Extremely \| Moderately \| Mildly \| Not at all \| \| --- \| --- \| --- \| --- \| |
| To what extent are you able to take care of all your personal needs (such as bathing and feeding yourself)? | \| Always \| Often \| Rarely \| Never \| \| --- \| --- \| --- \| --- \| |
| To what extent are you able to complete household and domestic tasks (such as getting groceries and managing your money)? | \| Always \| Often \| Rarely \| Never \| \| --- \| --- \| --- \| --- \| |
| Access Domain | |
| OUTPATIENT CARE: Within identified tools, the use of outpatient care was examined as an indicator of complexity. Data collection should include whether or not a patient is able to access primary health care, the number of visits they average in a year, and the types of specialists they are seeing (if any) for their health. | |
| Do you have a primary health care provider? | \| Yes \| No \| \| --- \| --- \| |
| How many doctors’ visits have you had in the past year? | \| 0 \| 1-3 \| 4-6 \| 7+ \| \| --- \| --- \| --- \| --- \| |
| Are you seeing any specialist doctors?  If yes, what types of specialists? | \| Yes \| No \| \| --- \| --- \|   Enter text: ________________ |
| HOSPITAL USE: Identified tools included items that addressed the frequency of hospital visits made by a patient. Data should also be collected on how many times a patient was admitted and how many times a patient sought care in the emergency department. | |
| How many times have you been admitted to the hospital for your health condition(s)? | \| 0 \| 1-3 \| 4-6 \| 7+ \| \| --- \| --- \| --- \| --- \| |
| How many times in the past year have you visited the ER for your health condition(s)? | \| 0 \| 1-3 \| 4-6 \| 7+ \| \| --- \| --- \| --- \| --- \| |
| COORDINATION OF CARE: Having adequate coordination of care was identified as an important aspect of managing and preventing complexity. Assessments of complexity should include the extent to which the patient feels the current care they are receiving is sufficient to manage their health. | |
| Do you feel that the coordination of care you are receiving now is effective for your health needs? | \| Yes \| No \| \| --- \| --- \| |
| Adverse Experiences Domain | |
| ADVERSITIES: Identified tools did not include items to assess how past negative life events and experiences have shaped the health of the patient, yet having adverse life experiences was a reality for many Indigenous patients as determined by our data collection. Assessments of patient complexity should include the extent to which one has experienced negative life events that may be contributing to their health to date. | |
| Have any of these things been a worry for you or anyone else living in this house during the last year?  Serious illness  Serious accident  Death of family member or close friend  Divorce or separation  Not able to get a job  Lost job  Alcohol related problems  Drug related problems  Seeing fights or people beaten up  Abuse or violent crime  Trouble with the police  Gambling problem  Member of family sent to jail  Overcrowding at home  Discrimination/Racism  Vandalism or Malicious damage to property | \| Yes \| No \| \| --- \| --- \| |
| How has your childhood history shaped your health and wellbeing today? | Enter text: ________________ |
| Healthcare Violence Domain | |
| HEALTHCARE VIOLENCE: Assessments of healthcare violence were missing from identified tools yet it emerged as a major cause of complexity as determined by our data collection. Assessments of Indigenous patient complexity should include the extent to which the patient feels their providers and/or systems are capable of addressing their needs in a safe and ethical space. | |
| Feeling that my doctor doesn't give me clear enough directions on how to manage my conditions | \| Not a problem \| A slight problem \| A moderate problem \| Somewhat serious problem \| A serious problem \| A very serious problem \| \| --- \| --- \| --- \| --- \| --- \| --- \| |
| Feeling that I don't have a doctor who I can see regularly enough about my conditions | \| Not a problem \| A slight problem \| A moderate problem \| Somewhat serious problem \| A serious problem \| A very serious problem \| \| --- \| --- \| --- \| --- \| --- \| --- \| |
| Feeling that I will end up with serious long-term complications, no matter what I do. | \| Not a problem \| A slight problem \| A moderate problem \| Somewhat serious problem \| A serious problem \| A very serious problem \| \| --- \| --- \| --- \| --- \| --- \| --- \| |
| Feeling that my doctor doesn't know enough about my conditions and its care | \| Not a problem \| A slight problem \| A moderate problem \| Somewhat serious problem \| A serious problem \| A very serious problem \| \| --- \| --- \| --- \| --- \| --- \| --- \| |
| Feeling that my doctor doesn't take my concerns seriously enough | \| Not a problem \| A slight problem \| A moderate problem \| Somewhat serious problem \| A serious problem \| A very serious problem \| \| --- \| --- \| --- \| --- \| --- \| --- \| |
| To what extent do you feel like you've been helped by previous healthcare providers? | \| Extremely \| Moderately \| Mildly \| Not at all \| \| --- \| --- \| --- \| --- \| |
| Do you feel like you can trust the healthcare system to treat you well and address your health needs? | \| Extremely \| Moderately \| Mildly \| Not at all \| \| --- \| --- \| --- \| --- \| |
| Do you feel safe when accessing healthcare services? | \| Extremely \| Moderately \| Mildly \| Not at all \| \| --- \| --- \| --- \| --- \| |
| Resilience and Culture Domain | |
| RESILIENCE AND CULTURE: Identified tools did not include assessments of resilience or any strengths-based aspects. From our data collection, resilience, strengths, and connection to one's culture were identified as “protective factors” that help to mitigate health complexity. Assessments should include inquiries into one's level of resilience and connection with culture to highlight a strengths-based approach to addressing complexity. | |
| I feel a strong attachment towards my [Aboriginal/FNMI] community or Nation | \| Strongly disagree \| Disagree \| Neither \| Agree \| Strongly agree \| \| --- \| --- \| --- \| --- \| --- \| |
| I have a strong sense of belonging to my [Aboriginal/FNMI] community or Nation | \| Strongly disagree \| Disagree \| Neither \| Agree \| Strongly agree \| \| --- \| --- \| --- \| --- \| --- \| |
| I feel a strong connection to my ancestors | \| Strongly disagree \| Disagree \| Neither \| Agree \| Strongly agree \| \| --- \| --- \| --- \| --- \| --- \| |
| I can understand some of my [Aboriginal/FNMI] language | \| Strongly disagree \| Disagree \| Neither \| Agree \| Strongly agree \| \| --- \| --- \| --- \| --- \| --- \| |
| I have participated in a cultural ceremony (examples: Sweatlodge, Moon Ceremony, Sundance, Longhouse, Feast or Giveaway) | \| Strongly disagree \| Disagree \| Neither \| Agree \| Strongly agree \| \| --- \| --- \| --- \| --- \| --- \| |
| I have a traditional person, Elder or Clan Mother who I talk to | \| Strongly disagree \| Disagree \| Neither \| Agree \| Strongly agree \| \| --- \| --- \| --- \| --- \| --- \| |
| When I am physically ill, I look to my [Aboriginal/FNMI] culture for help | \| Strongly disagree \| Disagree \| Neither \| Agree \| Strongly agree \| \| --- \| --- \| --- \| --- \| --- \| |
| When I am overwhelmed with my emotions, I look to my [Aboriginal/FNMI] culture for help | \| Strongly disagree \| Disagree \| Neither \| Agree \| Strongly agree \| \| --- \| --- \| --- \| --- \| --- \| |
| Do you feel like you have a balanced state of health? | \| Yes \| No \| \| --- \| --- \| |
| Are you able to connect with your culture? If not, do you want to? | \| Yes \| No \| \| --- \| --- \|  \| Yes \| No \| \| --- \| --- \| |
| What are your goals in terms of your health? How are you willing to achieve those goals? | Enter text: ________________  Enter text: ________________ |

**The following section is for the provider to complete:**

| Biological Domain |  |
| --- | --- |
| PROGNOSIS: Prognosis questions were included as part of identified tools yet they did not ask whether or not further investigations were required or if investigations have been done with little resolution for the patient. When addressing patient complexity, it is important to ask if all steps have been taken to identify the cause, or if despite taking all steps, there is still uncertainty regarding the cause of the patient’s health concerns. | |
| Thinking about your client’s physical health needs, are there any symptoms or problems (risk indicators) you are unsure about that require further investigation? | \| Definitely yes \| Probably yes \| Probably no \| Definitely no \| \| --- \| --- \| --- \| --- \| |
| Are further investigations required to understand the patient’s health concerns? | \| Yes \| No \| \| --- \| --- \| |
| Are there unexplained symptoms and/or signs despite having completed investigations and consultations? | \| Yes \| No \| \| --- \| --- \| |
| PRESENCE OF DISEASES: While we previously included a similar question for patients to list their own diseases and conditions, it is still important for the provider to indicate from their perspective which conditions and diseases are impacting the health of the patient. It is possible that the patient may not always be fully aware of their conditions or they may not accept them and subsequently not list them when asked. Therefore we included this concept in the provider's section as well. | |
| List all diagnoses currently impacting the patient | Enter text: ________________ |
| Social Domain | |
| DOMESTIC: Assessing one’s domestic environment, including physical and emotional safety within that environment, is an important social aspect that should be considered when investigating complexity. While the patient may report their home situation to be satisfactory, the provider may deem it to be unfit from their perspective. | |
| What is their home environment (including domestic violence, insecure housing, neighbor harassment)? | Enter text: ________________ |
| Literacy Domain | |
| GENERAL LITERACY: Identified tools inquired about a patient’s level of literacy as a potential source of complexity. The provider should take into consideration the patient's level of general literacy which is required for day-to-day interactions and tasks, including basic reading and writing skills. | |
| Is the patient able to read and write? | \| Yes \| No \| \| --- \| --- \| |
| HEALTH LITERACY: Health literacy was identified as being unique from general literacy and included the extent to which the patient is able to understand their health conditions and what they need to do in order to manage their health. | |
| How well do you perceive your patient understands their health and well-being (symptoms, signs or risk factors) and what they need to do to manage their health? | \| Extremely \| Moderately \| Mildly \| Not at all \| \| --- \| --- \| --- \| --- \| |
| Healthcare Access Domain | |
| COORDINATION OF CARE: Having adequate coordination of care was identified as an important aspect of managing and preventing complexity. From the perspective of the provider, coordination includes the extent to which they feel the current care being received by the patient is adequate (or not). | |
| Is the current coordination of care effective for the patient’s health needs? | \| Yes \| No \| \| --- \| --- \| |

**Appendix B.** Final framework concepts and items.

The following questions ask you about various factors that may be impacting your health and contributing to health complexity. Please answer each of the questions to the best of your ability so that your provider can get a “full picture” of what factors may be impacting your health.

| Biological Domain |  |
| --- | --- |
| GENDER IDENTITY AND EXPRESSION: | |
| Q1: Is your gender a source of complexity in your health and healthcare? | Enter text: ________________ |
| SYMPTOMS AND SELF-RATED HEALTH: | |
| Q2: To what degree do your medical condition(s) impact your level of functioning?  Explain how? | \| Extremely \| Moderately \| Mildly \| Not at all \| \| --- \| --- \| --- \| --- \|   Enter text: ________________ |
| PRESENCE OF DISEASES: | |
| Q3: List all conditions currently impacting you | Enter text: ________________ |
| PRESCRIPTIONS: | |
| Q4: What medications have been prescribed to you? | Enter text: ________________ |
| Q5: Do you have cost coverage for the medications that you should be taking? | \| Yes \| No \| \| --- \| --- \| |
| Q6: In your estimate, how many days of the week do you take your medication as prescribed? | Enter text: ________________ |
| Social Domain | |
| TRANSPORTATION: | |
| Q7: In terms of transportation, how do you get to the places that you need to go? | Enter text: ________________ |
| Q8: Do you have trouble accessing transportation? | \| Yes \| No \| \| --- \| --- \| |
| CAREGIVER: | |
| Q9: Do you require a caregiver?  If so, do you have one available to you? | \| Yes \| No \| \| --- \| --- \|  \| Yes \| No \| \| --- \| --- \| |
| Q10: Are you a caregiver for someone else? | \| Yes \| No \| \| --- \| --- \| |
| NETWORK: | |
| Q11: Do you have a social network that you participate in? | \| Yes \| No \| \| --- \| --- \| |
| Q12: Are you able to connect with the people closest to you when you want? | \| Always \| Often \| Rarely \| Never \| \| --- \| --- \| --- \| --- \| |
| Q13: How effective is your social network in promoting your health and wellness? | \| Extremely \| Moderately \| Mildly \| Not at all \| \| --- \| --- \| --- \| --- \| |
| HOUSING: | |
| Q14: Do you have a permanent address? | \| Yes \| No \| \| --- \| --- \| |
| Q15: What is your housing situation? | Enter text: ________________ |
| DOMESTIC: | |
| Q16: Are you physically safe in your home? | \| Yes \| No \| \| --- \| --- \| |
| Q17: Are you emotionally safe in your home? | \| Yes \| No \| \| --- \| --- \| |
| INCOME: | |
| Q18: What are your financial resources? | Enter text: ________________ |
| Q19: Do you have troubles making ends meet? | \| Yes \| No \| \| --- \| --- \| |
| Q20: To what extent is the money that you receive used to support others? | Enter text: ________________ |
| FOOD SECURITY: | |
| Q21: Do you often worry about how you will get your food? | \| Always \| Often \| Rarely \| Never \| \| --- \| --- \| --- \| --- \| |
| Psychological Domain | |
| MENTAL HEALTH: | |
| Q22: Do you feel like your mental health impacts your day-to-day life? | \| Yes \| No \| \| --- \| --- \| |
| Q23: To what extent do social stressors impact your mental health? | \| Extremely \| Moderately \| Mildly \| Not at all \| \| --- \| --- \| --- \| --- \| |
| EMOTIONS: | |
| Q24: How would you rate your emotional wellness? | \| Very poor \| Poor \| Good \| Excellent \| \| --- \| --- \| --- \| --- \| |
| Q25: To what extent have your emotions impacted your health and wellbeing? | \| Extremely \| Moderately \| Mildly \| Not at all \| \| --- \| --- \| --- \| --- \| |
| SUBSTANCE USE: | |
| Q25: Are you using any substances?  If yes, to what extent are these substances influencing your health? | \| Yes \| No \| \| --- \| --- \|  \| Extremely \| Moderately \| Mildly \| Not at all \| \| --- \| --- \| --- \| --- \| |
| Functioning Domain | |
| FUNCTIONING: | |
| Q27: To what extent do health issues impair your physical functioning? | \| Extremely \| Moderately \| Mildly \| Not at all \| \| --- \| --- \| --- \| --- \| |
| Q28: To what extent are you able to take care of all your personal needs (such as bathing and feeding yourself)? | \| Always \| Often \| Rarely \| Never \| \| --- \| --- \| --- \| --- \| |
| Q29: To what extent are you able to complete household and domestic tasks (such as getting groceries and managing your money)? | \| Always \| Often \| Rarely \| Never \| \| --- \| --- \| --- \| --- \| |
| Access Domain | |
| OUTPATIENT CARE: | |
| Q30: Do you have a primary health care provider? | \| Yes \| No \| \| --- \| --- \| |
| Q31: Approximately how many times have you visited a doctor and/or nurse practitioner in the past year? | \| 0 \| 1-3 \| 4-6 \| 7+ \| \| --- \| --- \| --- \| --- \| |
| Q32: Are you seeing any specialist doctors?  If yes, what types of specialists? | \| Yes \| No \| \| --- \| --- \|     Enter text: ________________ |
| HOSPITAL: | |
| Q33: Approximately how many times in the past year have you been admitted to the hospital for your health condition(s)? | \| 0 \| 1-3 \| 4-6 \| 7+ \| \| --- \| --- \| --- \| --- \| |
| Q34: Approximately how many times in the past year have you visited the ER or urgent care for your health condition(s)? | \| 0 \| 1-3 \| 4-6 \| 7+ \| \| --- \| --- \| --- \| --- \| |
| COORDINATION: | |
| Q35: Do you feel that the coordination of care you are receiving now is effective for your health needs? | \| Yes \| No \| \| --- \| --- \| |
| Adverse Experiences Domain | |
| ADVERSE EXPERIENCES: | |
| Q36: Have any of these things been a worry for you or anyone else living in this house during the last year?  Serious illness  Serious accident  Death of family member or close friend  Divorce or separation  Not able to get a job  Lost job  Alcohol related problems  Drug related problems  Seeing fights or people beaten up  Abuse or violent crime  Trouble with the police  Gambling problem  Member of family sent to jail  Overcrowding at home  Discrimination/Racism  Vandalism or Malicious damage to property | \| Yes \| No \| \| --- \| --- \| |
| Q37: How has your childhood history shaped your health and wellbeing today? | Enter text: ________________ |
| Healthcare Violence Domain | |
| HEALTHCARE VIOLENCE: | |
| Q38: Feeling that my doctor doesn't give me clear enough directions on how to manage my conditions | \| Not a problem \| A slight problem \| A moderate problem \| Somewhat serious problem \| A serious problem \| A very serious problem \| \| --- \| --- \| --- \| --- \| --- \| --- \| |
| Q39: Feeling that I don't have a doctor who I can see regularly enough about my conditions | \| Not a problem \| A slight problem \| A moderate problem \| Somewhat serious problem \| A serious problem \| A very serious problem \| \| --- \| --- \| --- \| --- \| --- \| --- \| |
| Q40: Feeling that I will end up with serious long-term complications, no matter what I do. | \| Not a problem \| A slight problem \| A moderate problem \| Somewhat serious problem \| A serious problem \| A very serious problem \| \| --- \| --- \| --- \| --- \| --- \| --- \| |
| Q41: Feeling that my doctor doesn't know enough about my conditions and its care | \| Not a problem \| A slight problem \| A moderate problem \| Somewhat serious problem \| A serious problem \| A very serious problem \| \| --- \| --- \| --- \| --- \| --- \| --- \| |
| Q42: Feeling that my doctor doesn't take my concerns seriously enough | \| Not a problem \| A slight problem \| A moderate problem \| Somewhat serious problem \| A serious problem \| A very serious problem \| \| --- \| --- \| --- \| --- \| --- \| --- \| |
| Q43: To what extent do you feel like you've been helped by previous healthcare providers? | \| Extremely \| Moderately \| Mildly \| Not at all \| \| --- \| --- \| --- \| --- \| |
| Q44: Do you feel like you can trust the healthcare system to treat you well and address your health needs? | \| Extremely \| Moderately \| Mildly \| Not at all \| \| --- \| --- \| --- \| --- \| |
| Q45: Do you feel safe when accessing healthcare services? | \| Extremely \| Moderately \| Mildly \| Not at all \| \| --- \| --- \| --- \| --- \| |
| Resilience and Culture Domain | |
| RESILIENCE AND CULTURE: | |
| Q46: I feel a strong attachment towards my [Aboriginal/FNMI] community or Nation | \| Strongly disagree \| Disagree \| Neither \| Agree \| Strongly agree \| \| --- \| --- \| --- \| --- \| --- \| |
| Q47: I have a strong sense of belonging to my [Aboriginal/FNMI] community or Nation | \| Strongly disagree \| Disagree \| Neither \| Agree \| Strongly agree \| \| --- \| --- \| --- \| --- \| --- \| |
| Q48: I feel a strong connection to my ancestors | \| Strongly disagree \| Disagree \| Neither \| Agree \| Strongly agree \| \| --- \| --- \| --- \| --- \| --- \| |
| Q49: I can understand some of my [Aboriginal/FNMI] language | \| Strongly disagree \| Disagree \| Neither \| Agree \| Strongly agree \| \| --- \| --- \| --- \| --- \| --- \| |
| Q50: I have participated in a cultural ceremony (examples: Sweatlodge, Moon Ceremony, Sundance, Longhouse, Feast or Giveaway) | \| Strongly disagree \| Disagree \| Neither \| Agree \| Strongly agree \| \| --- \| --- \| --- \| --- \| --- \| |
| Q51: I have a traditional person, Elder or Clan Mother who I talk to | \| Strongly disagree \| Disagree \| Neither \| Agree \| Strongly agree \| \| --- \| --- \| --- \| --- \| --- \| |
| Q52: When I am physically ill, I look to my [Aboriginal/FNMI] culture for help | \| Strongly disagree \| Disagree \| Neither \| Agree \| Strongly agree \| \| --- \| --- \| --- \| --- \| --- \| |
| Q53: When I am overwhelmed with my emotions, I look to my [Aboriginal/FNMI] culture for help | \| Strongly disagree \| Disagree \| Neither \| Agree \| Strongly agree \| \| --- \| --- \| --- \| --- \| --- \| |
| Q54: Do you feel like you have a balanced state of health? | \| Yes \| No \| \| --- \| --- \| |
| Q55: Are you able to connect with your culture?  If not, do you want to? | \| Yes \| No \| \| --- \| --- \|  \| Yes \| No \| \| --- \| --- \| |
| Q56: What are your goals in terms of your health?  How are you willing to achieve those goals? | Enter text: ________________  Enter text: ________________ |

**The following section is for the provider to complete:**

| Biological Domain |  |
| --- | --- |
| PROGNOSIS: | |
| Q57: Thinking about your client’s physical health needs, are there any symptoms or problems (risk indicators) you are unsure about that require further investigation? | \| Definitely yes \| Probably yes \| Probably no \| Definitely no \| \| --- \| --- \| --- \| --- \| |
| Q58: Are further investigations required to understand the patient’s health concerns? | \| Yes \| No \| \| --- \| --- \| |
| Q59: Are there unexplained symptoms and/or signs despite having completed investigations and consultations? | \| Yes \| No \| \| --- \| --- \| |
| PRESENCE OF DISEASES: | |
| Q60: List all diagnoses currently impacting the patient | Enter text: ________________ |
| Social Domain | |
| DOMESTIC: | |
| Q61: What is their home environment (including domestic violence, insecure housing, neighbor harassment)? | Enter text: ________________ |
| Literacy Domain | |
| GENERAL LITERACY: | |
| Q62: Is the patient able to read and write? | \| Yes \| No \| \| --- \| --- \| |
| HEALTH LITERACY: | |
| Q63: How well do you perceive your patient understands their health and well-being (symptoms, signs or risk factors) and what they need to do to manage their health? | \| Extremely \| Moderately \| Mildly \| Not at all \| \| --- \| --- \| --- \| --- \| |
| Access Domain | |
| COORDINATION: | |
| Q64: Is the current coordination of care effective for the patient’s health needs? | \| Yes \| No \| \| --- \| --- \| |
